# Supplementary figures and images for: Multiomic profiling of chronically activated CD4+ T cells identifies drivers of exhaustion and metabolic reprogramming
Source: PLoS Biol. 2024 Dec 17;22(12):e3002943. doi: 10.1371/journal.pbio.3002943 (PMC11703073; doi:10.1371/journal.pbio.3002943)

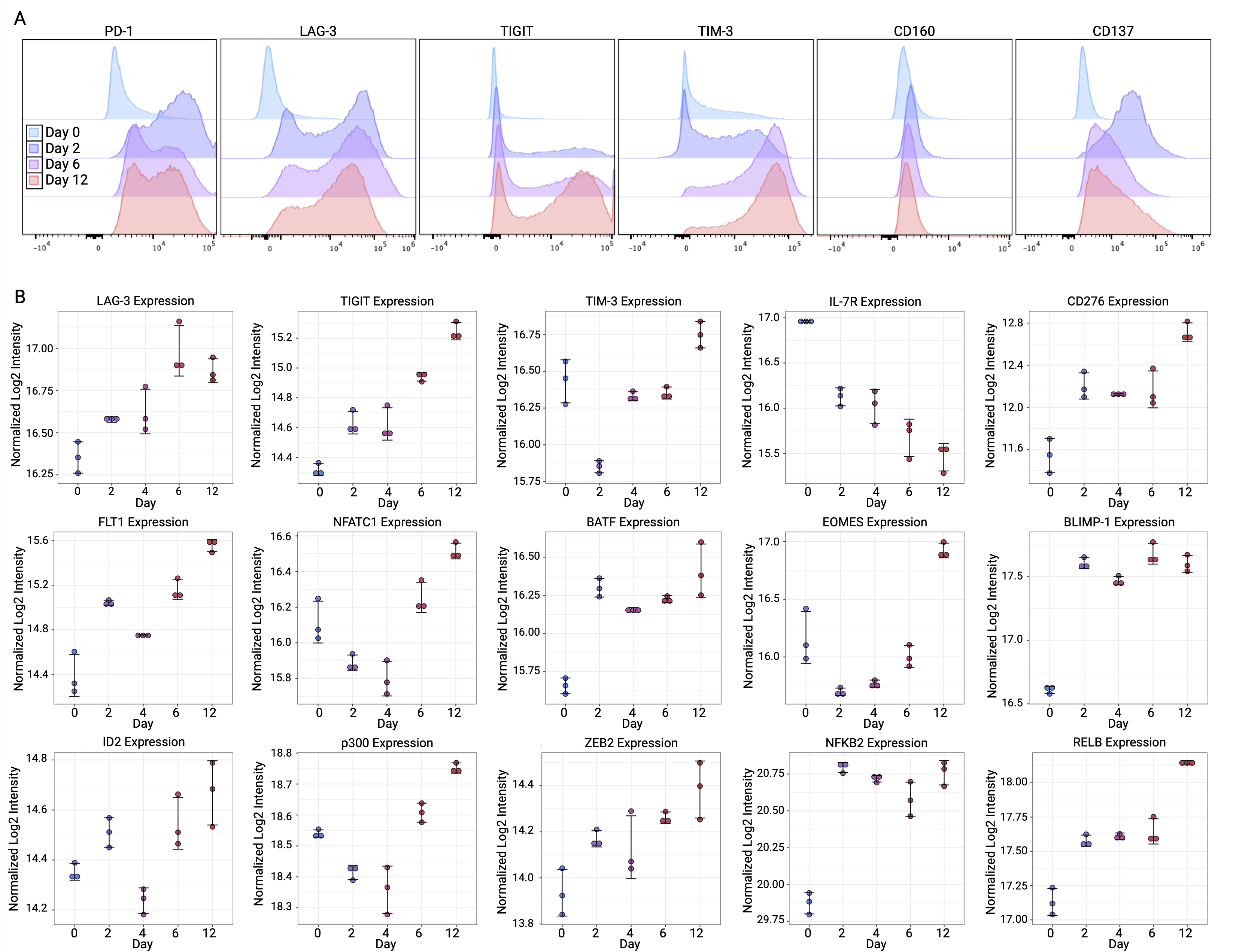

Supplement: S1 Fig — (A) Flow cytometric analysis of PD-1, LAG-3, TIGIT, TIM-3, CD160, and CD137 of time points (day 0, 2, 6, and 12) of a second donor CD4+ memory T cells brought through the in vitro exhaustion protocol. (B) Proteomic expression of exhaustion factors and selected novel factors to match those shown in main text. The data underlying this figure can be found in the supplementary flow files uploaded to flowrepository.org (A), and in S2 Data (B). Figure created with BioRender.com. (TIFF) [file pbio.3002943.s001.tiff]

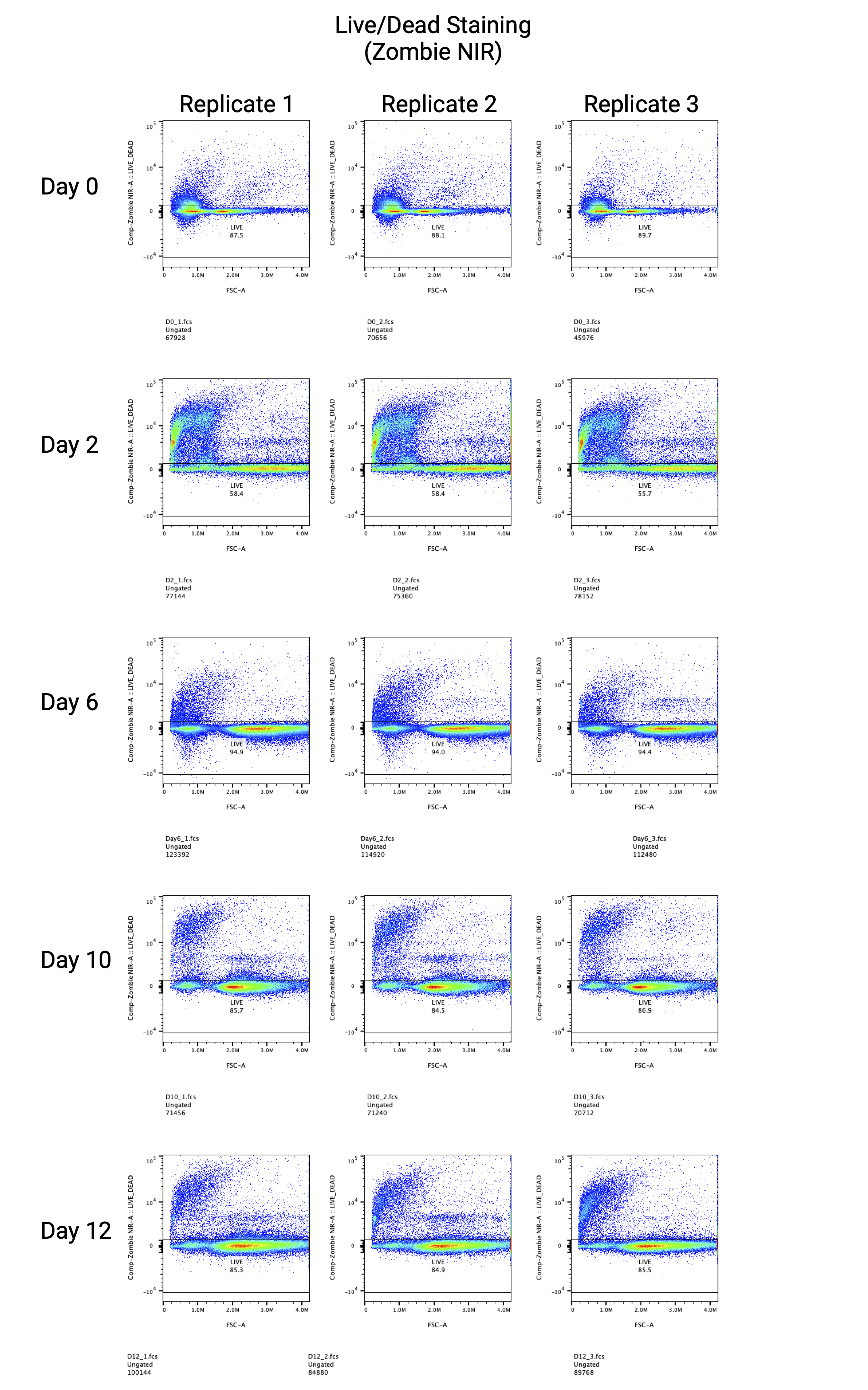

Supplement: S2 Fig — Flow cytometric analysis of donor 2 CD4+ cells at different time stages showing viability via a Live/Dead stain using Zombie NIR to stain dead/dying cells. Each time point was performed in triplicate cell cultures. The data underlying this figure can be found in the supplemental flow cytometry files uploaded to flowrepository.org. Figure created with BioRender.com. (TIFF) [file pbio.3002943.s002.tiff]

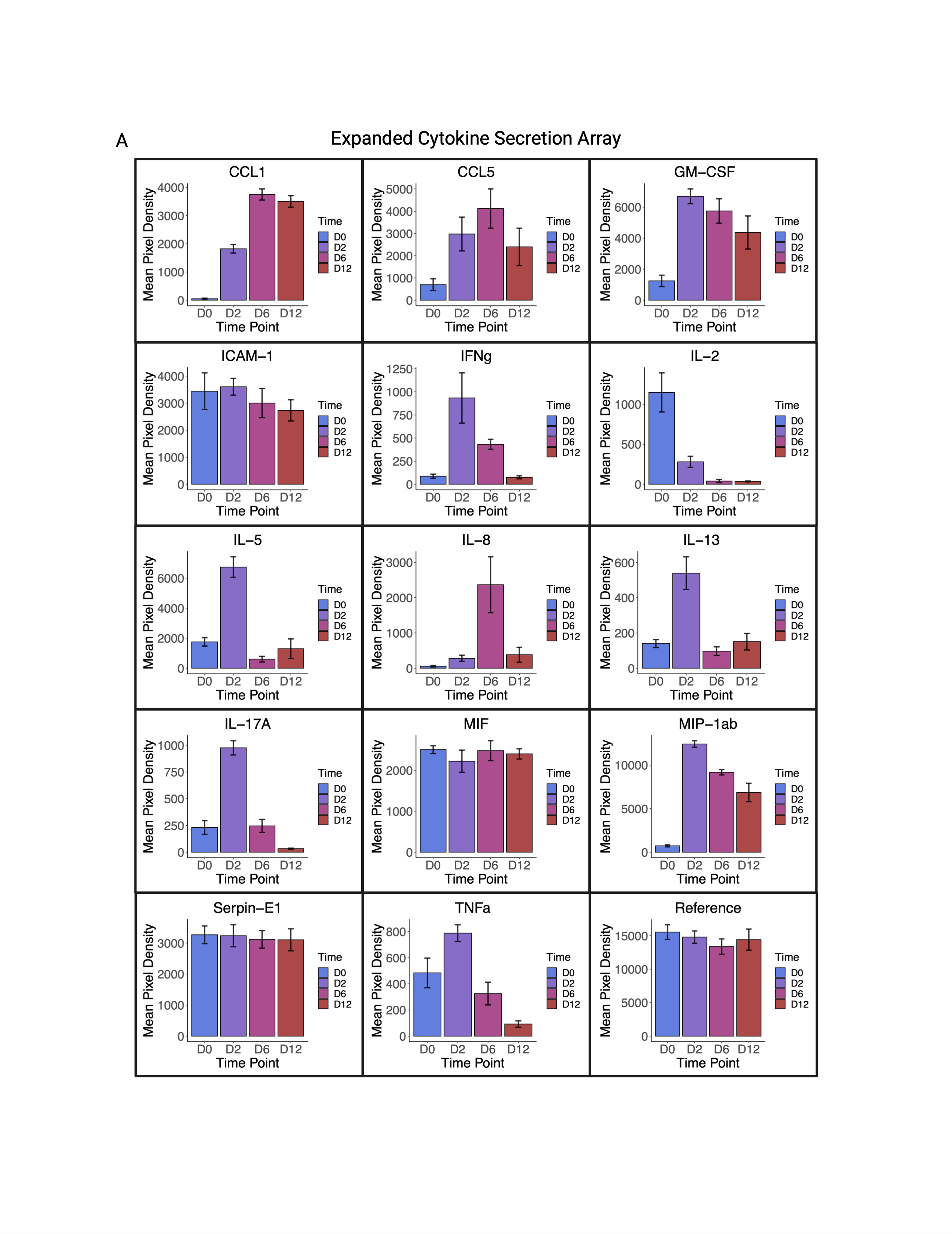

Supplement: S3 Fig — (A) Immunoblots of conditioned media of 14 cytokines commonly secreted by immune cells. Cells from donor #2 were brought through the exhaustion protocol, and conditioned media was made and collected at day 0, 2, 6, and 12 using methods described for cytokine release assay. Figure created with BioRender.com. (TIFF) [file pbio.3002943.s003.tiff]

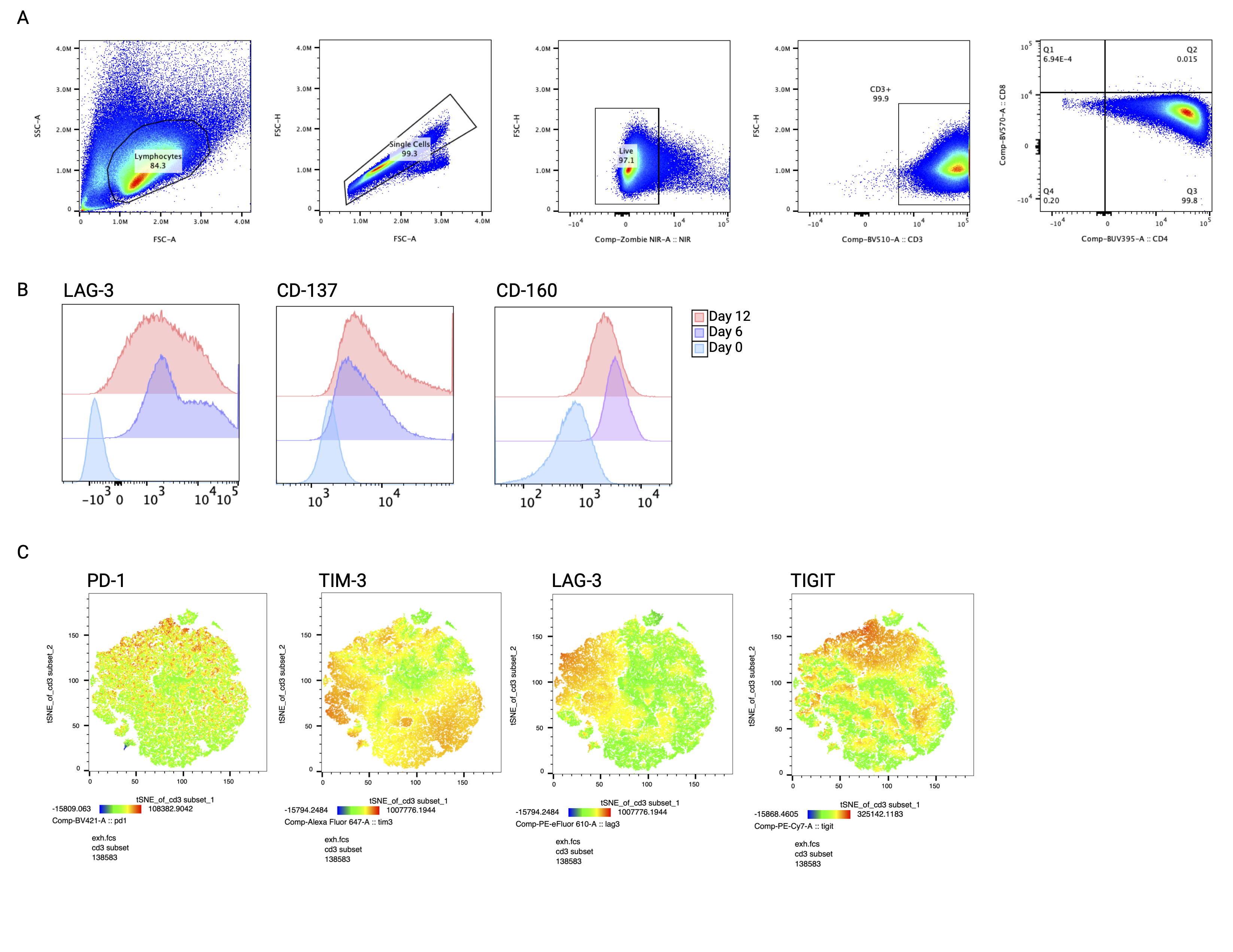

Supplement: S4 Fig — (A) Example (day 10) for gating strategy used for inhibitory receptor flow cytometry plots in Fig 1. (B) Flow cytometry analysis of LAG-3, CD-137, and CD-160 on day 0, day 6, and day 12 of chronic stimulation time course. (C) tSNE plots showing relative expression across cell population. Different donor was used here, and cells were analyzed on day 11 of exhaustion protocol. The data underlying this figure can be found in the supplemental flow cytometry files uploaded to flowrepository.org. Figure created with BioRender.com. (TIFF) [file pbio.3002943.s004.tiff]

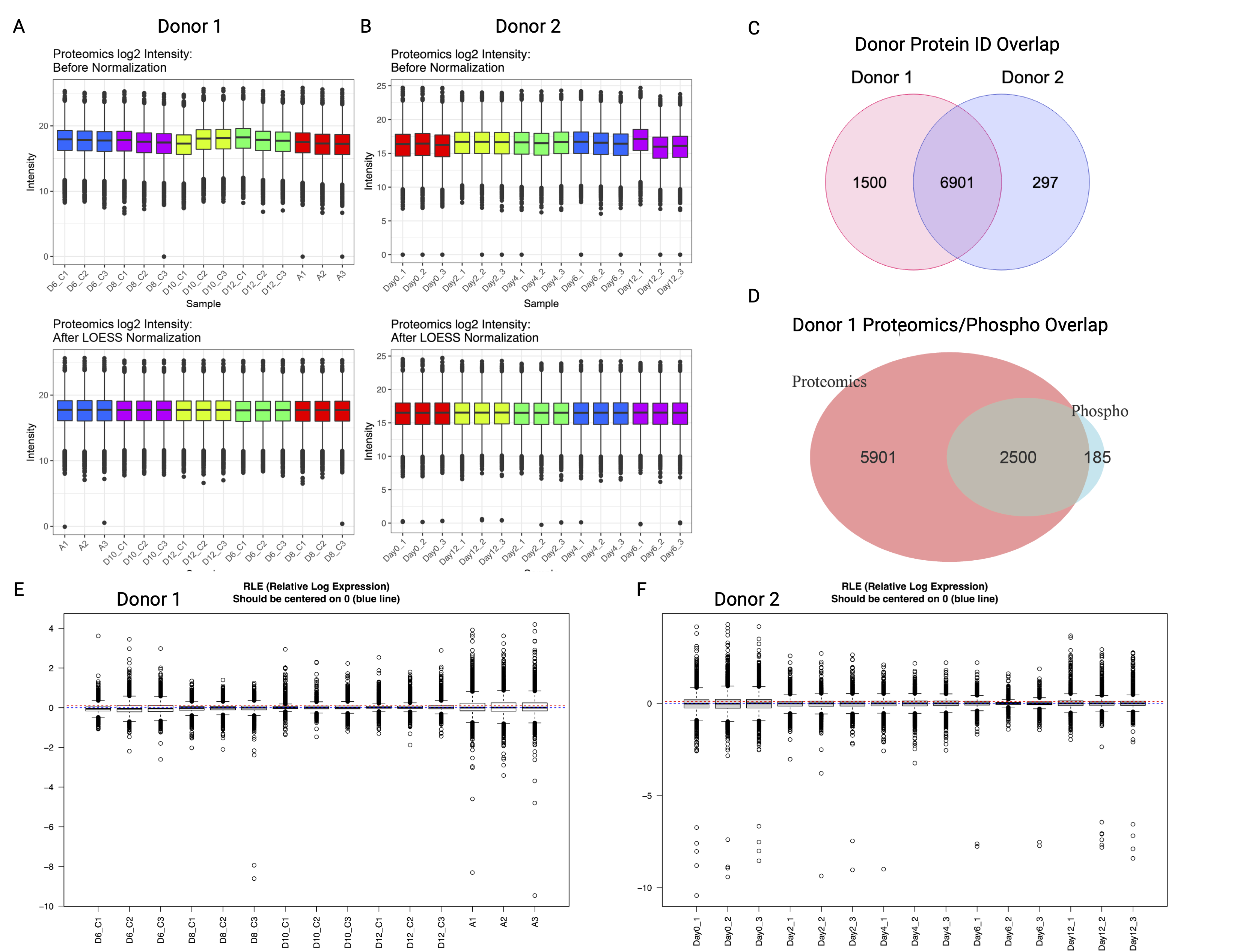

Supplement: S5 Fig — Intensity values of proteins in each sample, before and after loess normalization for (A) first donor and (B) second donor. (C) Venn diagram showing Donor 1 and 2 protein overlap. (D) Venn diagram showing number of proteins identified in proteomics and phosphoproteomics datasets and their overlap from donor 1. RLE plots for all samples for (E) donor 1 and (F) donor 2. The data underlying this figure can be found in S1 Data (A, E) and S2 Data. Figure created with BioRender.com. (TIFF) [file pbio.3002943.s005.tiff]

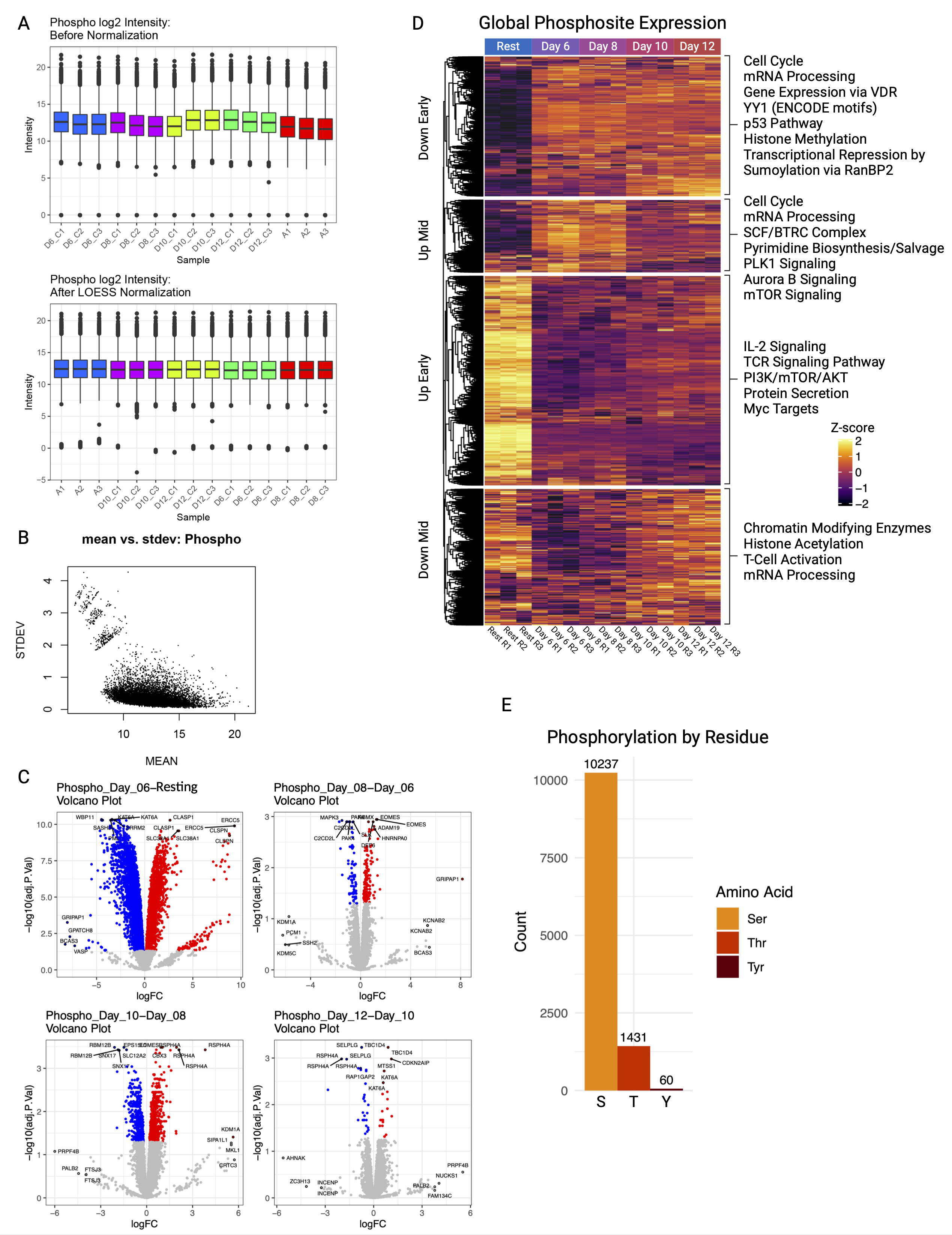

Supplement: S6 Fig — (A) Intensity values of phosphopeptides in each sample, before and after loess normalization. (B) Phosphopeptides mean vs. standard deviation of intensity values. (C) Volcano plots showing differential analysis of day 6 vs. resting, day 8 vs. day 6, day 10 vs. day 8, and day 12 vs. day 10. (D) Global phosphosite expression heatmap with phosphosites clustered based on expression profiles showing pathway enrichment by gene cluster. (E) Number of phosphorylation sites identified sorted by residue. The data underlying this figure can be found in S1 Data. Figure created with BioRender.com. (TIFF) [file pbio.3002943.s006.tiff]

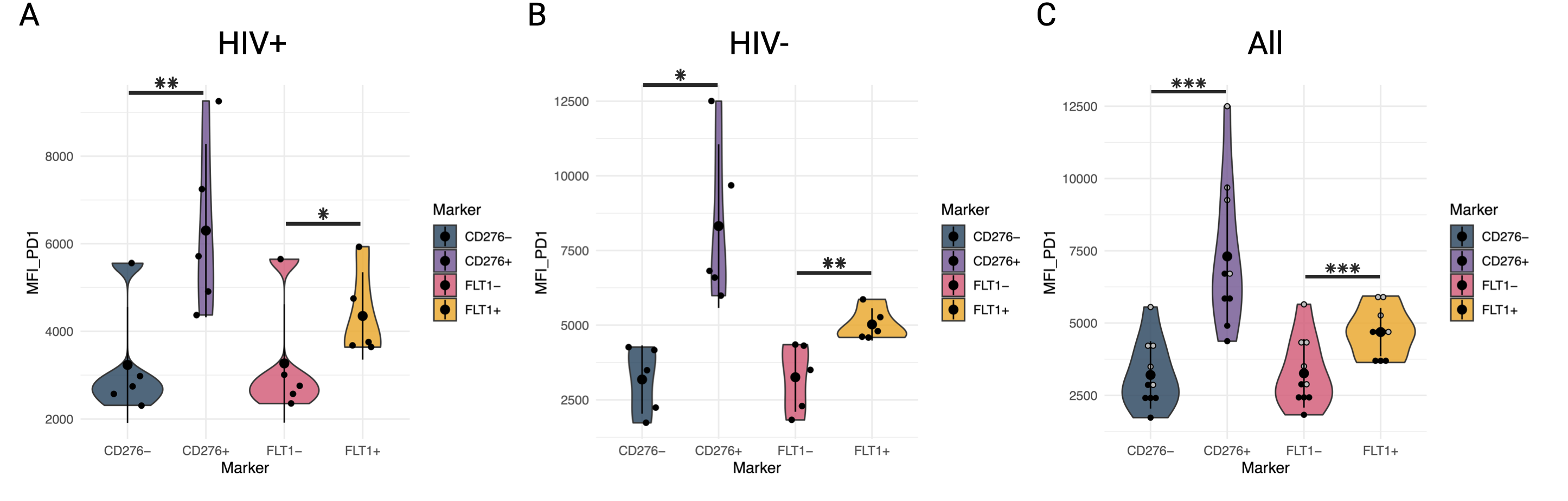

Supplement: S7 Fig — Mean fluorescence intensity (MFI) of PD-1 in CD4+ T cells from (A) HIV+, (B) HIV-, or (C) both population combined for CD4+ cells that are CD276+ or FLT-1+. The data from this figure can be found in the supplemental flow cytometry files uploaded to flowrepository.org. Figure created with BioRender.com. (TIFF) [file pbio.3002943.s007.tiff]

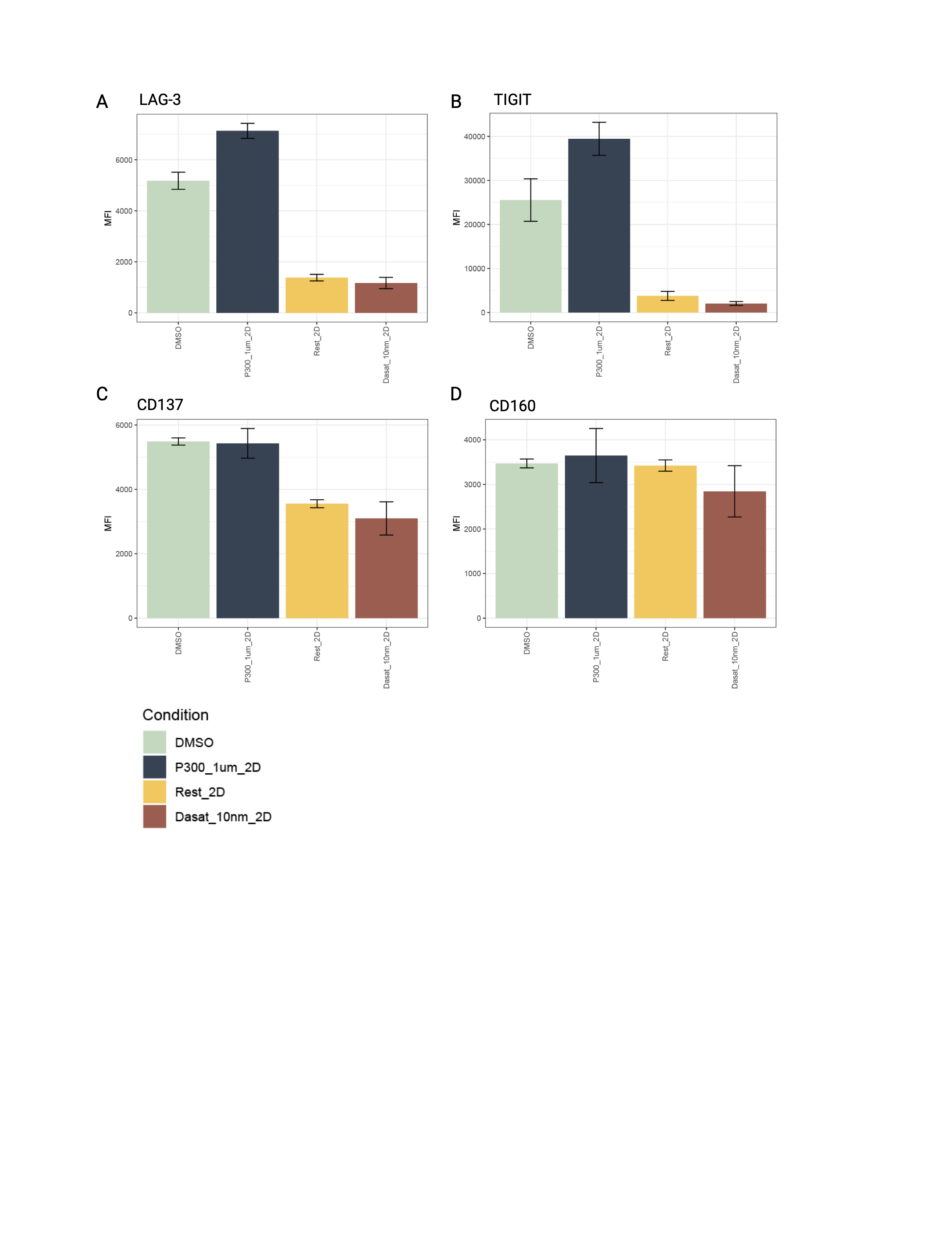

Supplement: S8 Fig — Mean fluorescence intensity (MFI) of (A) LAG-3, (B) TIGIT, (C) CD137, (D) CD160, in cells treated with vehicle (DMSO), p300 inhibitor (A-485), Dasatinib, or rested for 2 days. The data underlying this figure can be found in S3 Data. Figure created with BioRender.com. (TIFF) [file pbio.3002943.s008.tiff]

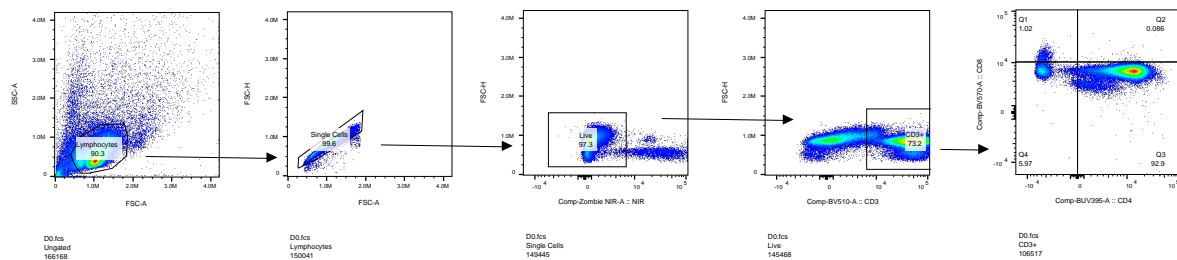

Supplement: S1 File — (ZIP) [file pbio.3002943.s021.zip › S1_File/Fig1_Gating_Strategy_Updated.pdf]

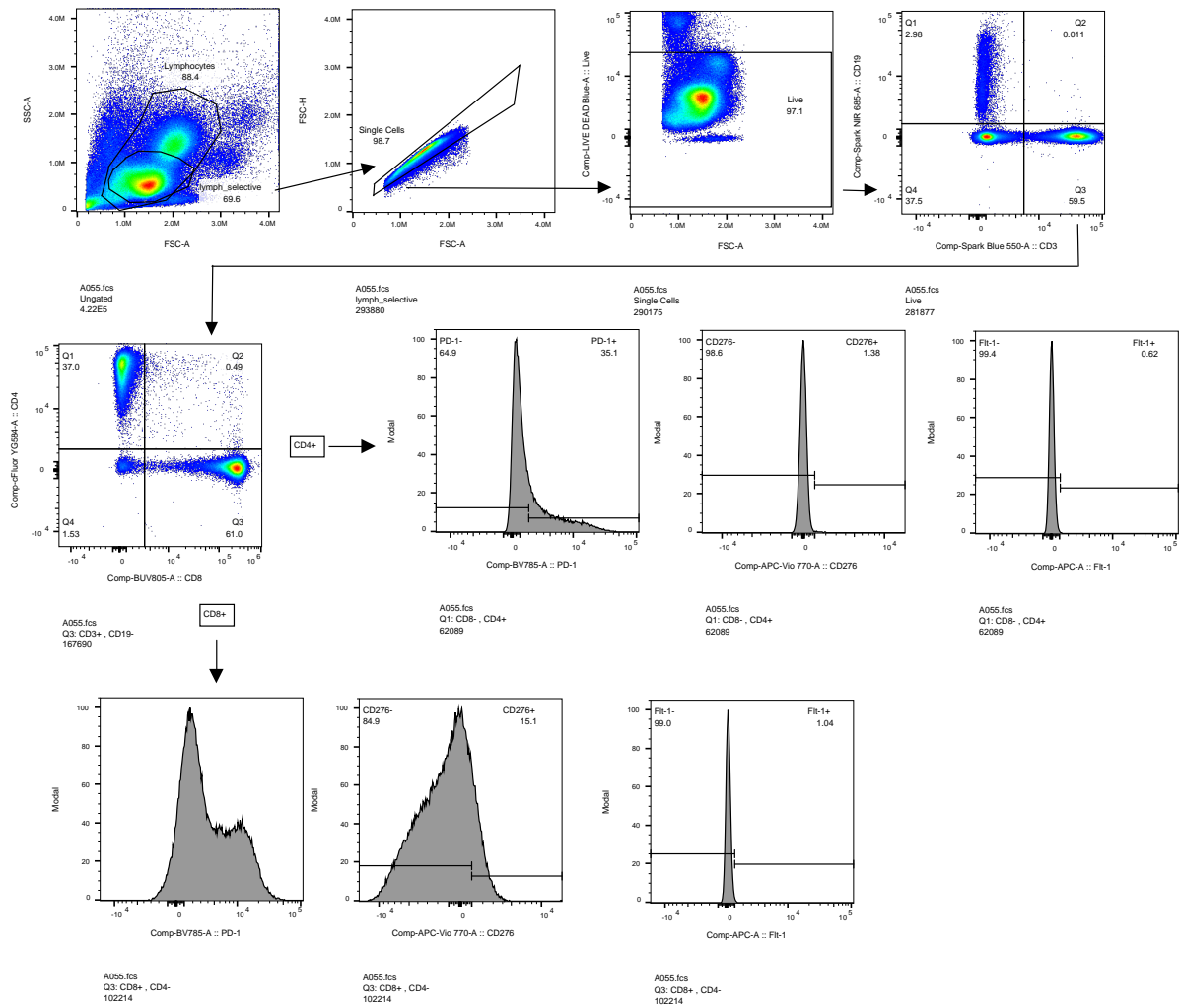

Supplement: S1 File — (ZIP) [file pbio.3002943.s021.zip › S1_File/Fig4_S7_Gating_Strategy.pdf]

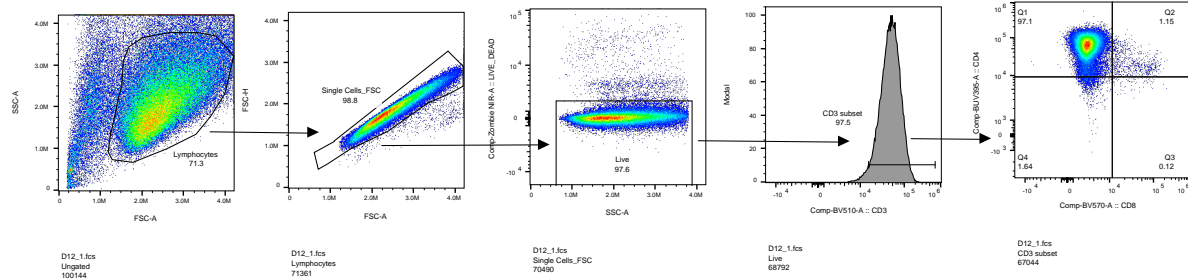

Supplement: S1 File — (ZIP) [file pbio.3002943.s021.zip › S1_File/FigS1_S2_S4_Gating_Strat_Updated.pdf]
