# Supplementary material for: Multiomic profiling of chronically activated CD4+ T cells identifies drivers of exhaustion and metabolic reprogramming
Source: PLoS Biol. 2024 Dec 17;22(12):e3002943. doi: 10.1371/journal.pbio.3002943 (PMC11703073; doi:10.1371/journal.pbio.3002943)
Supplement: Attachment — Submitted filename: Response_to_Reviewers.pdf [file pbio.3002943.s023.pdf]

Point-by-Point Responses to Reviewer Critiques

Reviewer #1:

"While the manuscript has its merits, several major issues are dampening my enthusiasm. Firstly, this study solely used one in vitro assay, which is not a well-accepted approach to study T cell exhaustion in the field."

We thank the reviewer for their knowledgeable and constructive feedback. The work shown in our manuscript could not have been performed without such an assay since one cannot isolate sufficient numbers of exhausted cells from patients for deep multi-omic analysis. We also recognize that this model has caveats, and may not fully reflect the exhaustion state seen *in vivo*, and so have now added extensive additional validation experiments, including flow cytometric analysis for novel cell surface markers identified in our manuscript, proteomic analysis of pooled HIV+ donor T cells, and comparison of proteomic results to single-cell RNA sequencing of both CD4+ and CD8+ tumor infiltrating lymphocytes.

Our intent is to provide the community with reliable datasets for follow up studies, and note that our model/protocol has in fact been used/cited by multiple other researchers in the field (see references 1-5 below).

1. Balkhi, M. Y., Wittmann, G., Xiong, F., & Junghans, R. P. (2018). YY1 Upregulates Checkpoint Receptors and Downregulates Type I Cytokines in Exhausted, Chronically Stimulated Human T Cells. *iScience*, *2*, 105–122. https://doi.org/10.1016/j.isci.2018.03.009
2. Simula, L., Antonucci, Y., Scarpelli, G., Cancila, V., Colamatteo, A., Manni, S., De Angelis, B., Quintarelli, C., Procaccini, C., Matarese, G., Tripodo, C., & Campello, S. (2022). PD-1-induced T cell exhaustion is controlled by a Drp1-dependent mechanism. *Molecular Oncology*, *16*(1), 188–205. https://doi.org/10.1002/1878-0261.13103
3. Zhao, M., Kiernan, C. H., Stairiker, C. J., Hope, J. L., Leon, L. G., Meurs, M. van, Brouwers-Haspels, I., Boers, R., Boers, J., Gribnau, J., IJcken, W. F. J. van, Bindels, E. M., Hoogenboezem, R. M., Erkeland, S. J., Mueller, Y. M., & Katsikis, P. D. (2020). Rapid in vitro generation of bona fide exhausted CD8+ T cells is accompanied by Tcf7 promotor methylation. *PLOS Pathogens*, *16*(6), e1008555. https://doi.org/10.1371/journal.ppat.1008555
4. Zhu, L., Zhou, X., Gu, M., Kim, J., Li, Y., Ko, C.-J., Xie, X., Gao, T., Cheng, X., & Sun, S.-C. (2022). Dapl1 controls NFATc2 activation to regulate CD8+ T cell exhaustion and responses in chronic infection and cancer. *Nature Cell Biology*, *24*(7), Article 7. https://doi.org/10.1038/s41556-022-00942-8
5. Dunsford, L.S., Thoirs, R.H., Rathbone, E., Patakas, A. (2020). A Human In Vitro T Cell Exhaustion Model for Assessing Immuno-Oncology Therapies. In: Tan, SL. (eds) Immuno-Oncology. Methods in Pharmacology and Toxicology. Humana, New York, NY. https://doi.org/10.1007/978-1-0716-0171-6_6

"Secondly, the authors failed to demonstrate the cells generated from this in vitro assay resemble 'dysfunctional' CD4 T cells in human diseases."

While not studied to the same level as CD8 cells (part of the rationale for our study), there is ample prior evidence that CD4 T cells have an analogous exhausted state: Upregulation of inhibitory receptors and exhaustion markers (just as we report) have been documented on CD4+ T cells during the classic exhaustion model of LCMV (1, 2 below) and other chronic/recurrent infections (3-5). Similar to CD8+ T cells, upon repeated antigen exposure CD4+ T cells in infected tissues have elevated inhibitory markers (6, 7); the expression levels of these markers is correlated with disease severity (8-12). Prior studies have also reported concomitant loss of CD4+ effector functions, key hallmarks of exhaustion, including impaired cytokine production (2, 13), reduced splenic motility (14), and reduced response to

secondary challenge (13). In agreement with our data, chronic HIV infection of CD4 cells *in vivo* show elevated granzyme B (15) and perforin (16), as seen in terminally exhausted CD8+ T cells (17).

Nevertheless, to directly address this important caveat, we examined the expression of exhaustion markers and novel factors identified in our in vitro analysis in two manners: First, by systematically surveying a recent in vivo single-cell sequencing study of breast tumor resident CD4+ (and CD8+) T cells (18). Strikingly, as with the dysfunctional CD8+ state, we found that a major subpopulation of tumor infiltrating CD4+ T cells display elevated expression of inhibitory factors *in patient tissue* (see below), and now described in our revised manuscript **Figure 7**, including prominent markers (PD-1, LAG-3, TIGIT), key transcription factors (TOX, PRDM1, NFATC1), and many of the novel candidates found using our in vitro model (p300, ZEB2, VDR, etc).

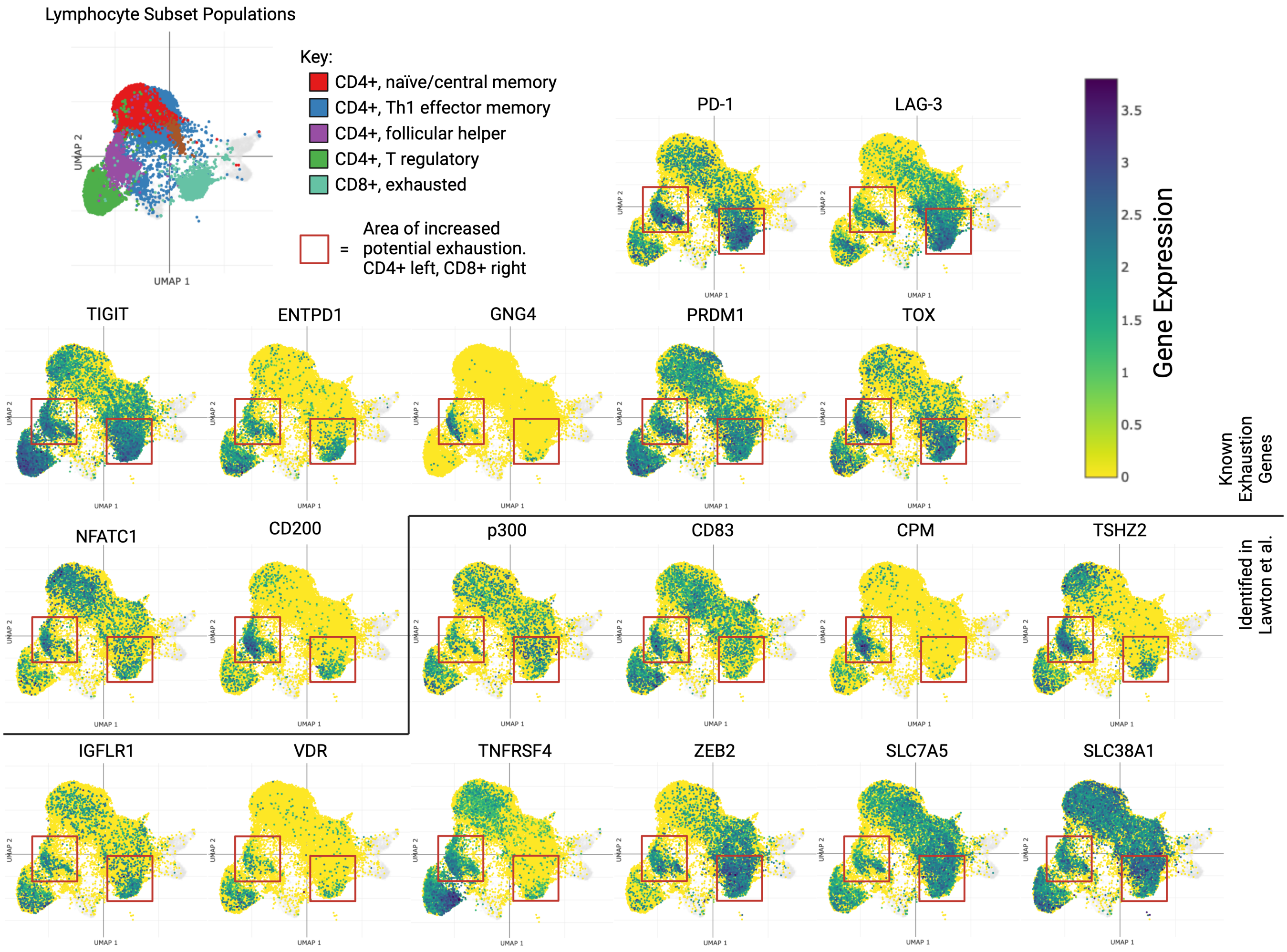

Figure 7. Gene expression of exhaustion markers and novel proteins identified in exhaustion model in a recently published breast cancer single-cell sequencing data set (18 below, 63 in manuscript). UMAP showing CD4+ and CD8+ T-cell subtypes showing expression of (A) known exhaustion-associated genes, and (B) newly reported factors identified in the model presented by our study (Lawton et al).

1. Dow, C., Henderson, R., Sette, A., & Mothé, B. R. (2013). CD4+ T-cell inhibitory ligands: A tool for characterizing dysfunctional CD4+ T cells during chronic infection. *Immunology*, *140*(1), 61–69. https://doi.org/10.1111/imm.12109
2. Aubert, R. D., Kamphorst, A. O., Sarkar, S., Vezys, V., Ha, S.-J., Barber, D. L., Ye, L., Sharpe, A. H., Freeman, G. J., & Ahmed, R. (2011). Antigen-specific CD4 T-cell help rescues exhausted CD8 T cells during chronic viral infection. *Proceedings of the National Academy of Sciences*, *108*(52), 21182–21187. https://doi.org/10.1073/pnas.1118450109
3. Crawford, A., Angelosanto, J. M., Kao, C., Doering, T. A., Odorizzi, P. M., Barnett, B. E., & Wherry, E. J. (2014). Molecular and Transcriptional Basis of CD4+ T Cell Dysfunction during Chronic Infection. *Immunity*, *40*(2), 289–302. https://doi.org/10.1016/j.immuni.2014.01.005
4. Kasprowicz, V., Schulze Zur Wiesch, J., Kuntzen, T., Nolan, B. E., Longworth, S., Berical, A., Blum, J., McMahon, C., Reyor, L. L., Elias, N., Kwok, W. W., McGovern, B. G., Freeman, G., Chung, R. T., Klenerman, P., Lewis-Ximenez, L., Walker, B. D., Allen, T. M., Kim, A. Y., & Lauer, G. M. (2008). High level of PD-1 expression on hepatitis C virus (HCV)-specific CD8+ and CD4+ T cells during acute HCV infection, irrespective of clinical outcome. *Journal of Virology*, *82*(6), 3154–3160. https://doi.org/10.1128/JVI.02474-07
5. Noyan, K., Nguyen, S., Betts, M. R., Sönnerborg, A., & Buggert, M. (2018). Human Immunodeficiency Virus Type-1 Elite Controllers Maintain Low Co-Expression of Inhibitory Receptors on CD4+ T Cells. *Frontiers in Immunology*, *9*. https://www.frontiersin.org/articles/10.3389/fimmu.2018.00019
6. Barathan, M., Gopal, K., Mohamed, R., Ellegård, R., Saeidi, A., Vadivelu, J., Ansari, A. W., Rothan, H. A., Ravishankar Ram, M., Zandi, K., Chang, L. Y., Vignesh, R., Che, K. F., Kamarulzaman, A., Velu, V., Larsson, M., Kamarul, T., & Shankar, E. M. (2015). Chronic hepatitis C virus infection triggers spontaneous differential expression of biosignatures associated with T cell exhaustion and apoptosis signaling in peripheral blood mononucleocytes. *Apoptosis: An International Journal on Programmed Cell Death*, *20*(4), 466–480. https://doi.org/10.1007/s10495-014-1084-y
7. Nebbia, G., Peppa, D., Schurich, A., Khanna, P., Singh, H. D., Cheng, Y., Rosenberg, W., Dusheiko, G., Gilson, R., ChinAleong, J., Kennedy, P., & Maini, M. K. (2012). Upregulation of the Tim-3/Galectin-9 Pathway of T Cell Exhaustion in Chronic Hepatitis B Virus Infection. *PLOS ONE*, *7*(10), e47648. https://doi.org/10.1371/journal.pone.0047648
8. Antoine, P., Olislagers, V., Huygens, A., Lecomte, S., Liesnard, C., Donner, C., & Marchant, A. (2012). Functional Exhaustion of CD4+ T Lymphocytes during Primary Cytomegalovirus Infection. *The Journal of Immunology*, *189*(5), 2665–2672. https://doi.org/10.4049/jimmunol.1101165
9. Vingert, B., Benati, D., Lambotte, O., de Truchis, P., Slama, L., Jeannin, P., Galperin, M., Perez-Patrigeon, S., Boufassa, F., Kwok, W. W., Lemaître, F., Delfraissy, J.-F., Thèze, J., & Chakrabarti, L. A. (2012). HIV Controllers Maintain a Population of Highly Efficient Th1 Effector Cells in Contrast to Patients Treated in the Long Term. *Journal of Virology*, *86*(19), 10661–10674. https://doi.org/10.1128/jvi.00056-12
10. Kaufmann, D. E., Kavanagh, D. G., Pereyra, F., Zaunders, J. J., Mackey, E. W., Miura, T., Palmer, S., Brockman, M., Rathod, A., Piechocka-Trocha, A., Baker, B., Zhu, B., Le Gall, S., Waring, M. T., Ahern, R., Moss, K., Kelleher, A. D., Coffin, J. M., Freeman, G. J., … Walker, B. D. (2007). Upregulation of CTLA-4 by HIV-specific CD4+ T cells correlates with disease progression and defines a reversible immune dysfunction. *Nature Immunology*, *8*(11), Article 11. https://doi.org/10.1038/ni1515
11. Jones, R. B., Ndhlovu, L. C., Barbour, J. D., Sheth, P. M., Jha, A. R., Long, B. R., Wong, J. C., Satkunarajah, M., Schweneker, M., Chapman, J. M., Gyenes, G., Vali, B., Hyrcza, M. D., Yue, F. Y., Kovacs, C., Sassi, A., Loutfy, M., Halpenny, R., Persad, D., … Ostrowski, M. A. (2008). Tim-3 expression defines a novel population of dysfunctional T cells with highly elevated frequencies in progressive HIV-1 infection. *Journal of Experimental Medicine*, *205*(12), 2763–2779. https://doi.org/10.1084/jem.20081398
12. Wu, W., Shi, Y., Li, J., Chen, F., Chen, Z., & Zheng, M. (2011). Tim-3 expression on peripheral T cell subsets correlates with disease progression in hepatitis B infection. *Virology Journal*, *8*(1), 113. https://doi.org/10.1186/1743-422X-8-113
13. Brooks, D. G., Teyton, L., Oldstone, M. B. A., & McGavern, D. B. (2005). Intrinsic Functional Dysregulation of CD4 T Cells Occurs Rapidly following Persistent Viral Infection. *Journal of Virology*, *79*(16), 10514–10527. https://doi.org/10.1128/jvi.79.16.10514-10527.2005
14. Zinselmeyer, B. H., Heydari, S., Sacristán, C., Nayak, D., Cammer, M., Herz, J., Cheng, X., Davis, S. J., Dustin, M. L., & McGavern, D. B. (2013). PD-1 promotes immune exhaustion by inducing antiviral T cell motility paralysis. *Journal of Experimental Medicine*, *210*(4), 757–774. https://doi.org/10.1084/jem.20121416
15. Salwe, S., Singh, A., Padwal, V., Velhal, S., Nagar, V., Patil, P., Deshpande, A., & Patel, V. (2019). Immune signatures for HIV-1 and HIV-2 induced CD4+T cell dysregulation in an Indian cohort. *BMC Infectious Diseases*, *19*(1), 135. https://doi.org/10.1186/s12879-019-3743-7

16. Stone, S., Price, P., & French, M. (2005). Dysregulation of CD28 and CTLA-4 expression by CD4 T cells from previously immunodeficient HIV-infected patients with sustained virological responses to highly active antiretroviral therapy. *HIV Medicine*, *6*(4), 278–283. https://doi.org/10.1111/j.1468-1293.2005.00307.x
17. Miller, B. C., Sen, D. R., Al Abosy, R., Bi, K., Virkud, Y. V., LaFleur, M. W., Yates, K. B., Lako, A., Felt, K., Naik, G. S., Manos, M., Gjini, E., Kuchroo, J. R., Ishizuka, J. J., Collier, J. L., Griffin, G. K., Maleri, S., Comstock, D. E., Weiss, S. A., … Haining, W. N. (2019). Subsets of exhausted CD8+ T cells differentially mediate tumor control and respond to checkpoint blockade. *Nature Immunology*, *20*(3), Article 3. https://doi.org/10.1038/s41590-019-0312-6
18. Wu SZ, Al-Eryani G, Roden DL, Junankar S, Harvey K, Andersson A, et al. A single-cell and spatially resolved atlas of human breast cancers. Nat Genet 2021;53:1334–47.

“Thirdly, the authors did not dissect the effects of chronic stimulation on different Th subsets despite total memory CD4+ T cells were used in the study. Hence, the very heavy reliance on one assay for the conclusions of the paper are problematic and I do not think that the field will accept that conclusions in this model are representative of changes in "exhaustion".”

This is a great point for future studies, but beyond the scope and capability of our original intent. While studying the effects of chronic stimulation on different Th subsets would be interesting and insightful, we could not separate a sufficient numbers of T cells by multi-step isolation that would also meet the large cell counts required for our deep multi-omic profiling workflows. As mass spec technology and protein characterization efficiency advance, this should become more practical to perform in the future.

Key remaining concerns:

1. “As stated by the authors, the definition of T cell exhaustion is mainly derived from studies with CD8+T cells, and the existence of CD4 T cell exhaustion remains elusive. Yet, the authors referenced their findings to CD8 T cell exhaustion datasets and features. Given the fundamental differences in CD4 versus CD8 T cell biology, I am concerned about this approach. Instead, the authors should reference their findings to CD4 T cell datasets derived from cancer patients or patients with chronic infections. Do these in vitro- generate cells indeed resemble chronically activated dysfunctional CD4T cells in human diseases?”

This is an important point. As suggested by the reviewer and noted above, we have now performed a direct comparison to available CD4+ T cell cancer datasets. While it’s true that CD4+ and CD8+ T cells have different biology, strikingly many of the major hallmarks of CD8+ exhaustion are in fact seen in the chronically stimulated CD4+ tumor T cells in vivo (as shown above/**Fig 7**). We would like to emphasize that our initial finding and independent validation that the key hallmarks of CD8+ exhaustion are replicated in CD4+ cells, both in vitro and in vivo, is an important finding in itself.

2. “All memory T cells, including Tregs, Th1, Th2, Th17 cells, were used in this study. Would the 'exhaustion' features of these Th subsets be similar? Is it appropriate to use IFNg/TNFa/IL-2 production and inhibitory receptor expression to generalize ' exhaustion' of all CD4 T cell subsets?”

This is an excellent question and would make a great follow-up study, but beyond the scope of our initial multi-pronged study. For our deep profiling experiments, we could not separate suitable number of T cell subsets for our multi-omics workflows given the generally low numbers of cells one obtains from multi-step T-cell isolations. As technology and methods advance, this should become more practical to perform. As far as cytokines measured, we have now performed additional cytokine profiling experiments encompassing a total of 14 different cytokines, which all show the same consistent

patterns as reported before, which we now include both below and append to the updated manuscript as **Supplemental Figure 3**.

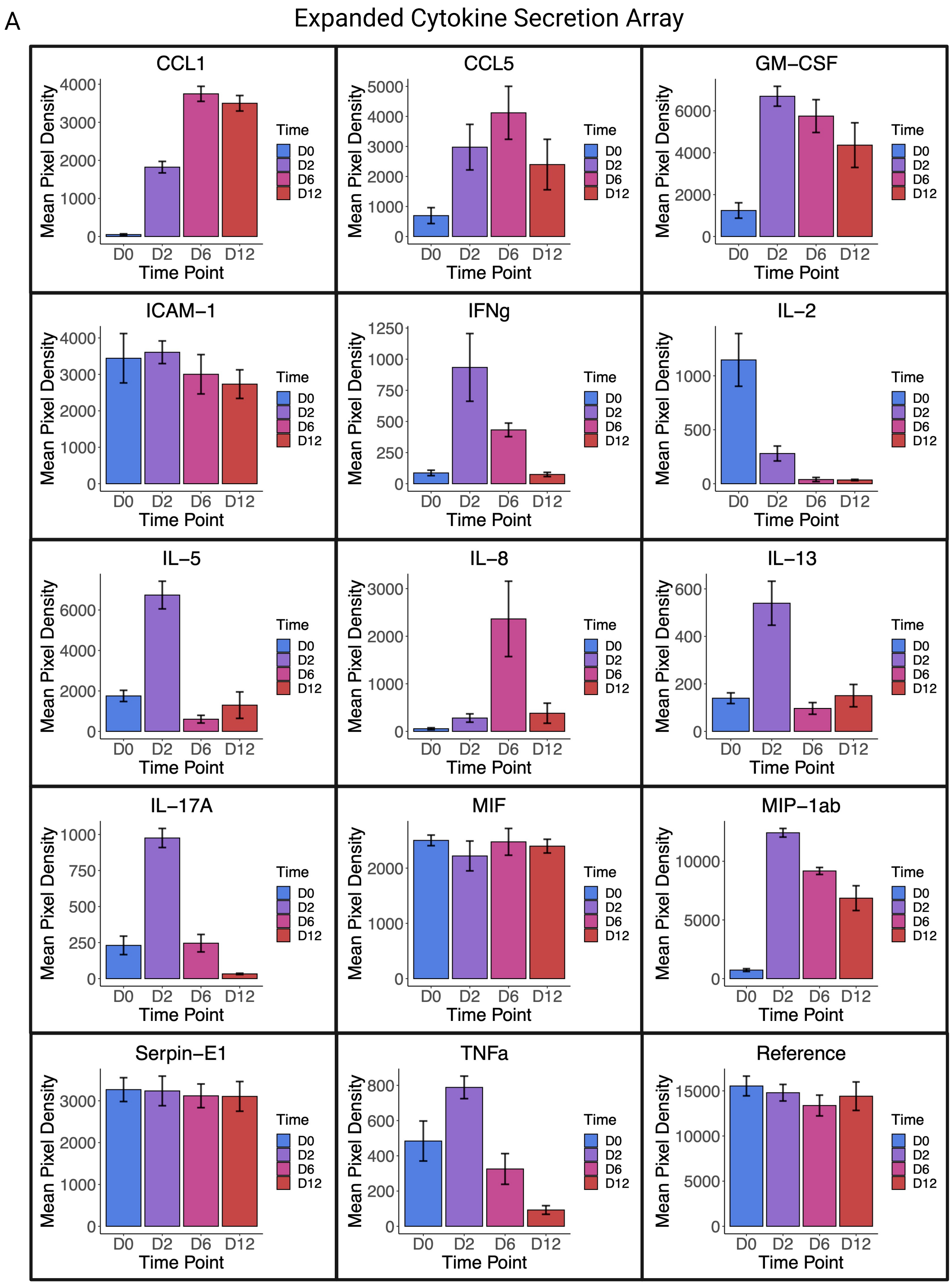

Supplemental Figure 3. Expanded secretion assays show altered cytokine production in chronically stimulated CD4+ memory cells. (A) Immunoblots of conditioned media of 14 different

cytokines reported to be secreted by immune cells. Cells (donor #2) were brought through the in vitro exhaustion protocol, and conditioned media was collected at day 0, 2, 6, and 12 using the same methods described for the original cytokine release assay.

3. "It is well known that the standard culture medium do not reflect physiological metabolite concentrations, leading to inaccurate results. Physiological media e.g. plasma-like media, should be used to confirm the metabolic findings."

While we concur that media does not recapitulate exact physiological conditions, the metabolomics literature commonly reports analyses of samples from cultured cells. The medium we employed is commonly used by many labs working with T-cells *ex vivo*. We also replaced fetal bovine serum with human serum to improve physiological relevance. We do recognize the limitations of our model, which we note at length in the updated Discussion ("Limitations of our study") but emphasize that our approach allowed us to study metabolism shifts independent of alterations in metabolite concentrations in the surrounding microenvironment, such as a tumor, which are nutrient scarce.

4. *"Metabolic assays showed [should] be performed to confirm the metabolic rewiring suggested in figure 4* [now Fig 6]*."*

We do not show metabolic rewiring in that figure, but assuming the reviewer is referring to figure 6 (what was figure 5, metabolomics), we respectfully counter that additional metabolic assays are not warranted since we not only predicted metabolic pathway changes from both our proteomic and phosphoproteomics data, but actually used untargeted metabolomics as an orthogonal method to directly observe metabolite changes that verify our pathway predictions.

5. *"Do the metabolic changes dictated by P300 network ? Could A-485 rescue the metabolic defects?"*

Interesting question. Whether p300 is involved in the observed metabolism changes is currently not known in T cells. Studies have shown that p300 mediates certain metabolism changes in other contexts, such as regulation of glycolysis or nutrient sensing, so this remains a good possibility which we plan to revisit in a future follow up study.

6. "What causes the reduction in IL-2 and TNFa production? What are the roles of P300 network and metabolic dysregulation in this regard?"

It is well documented that both IL-2 and TNFa become downregulated during T-cell exhaustion. As far as we are aware, the mechanism of their downregulation is not precisely known, but is likely controlled through the same major transcriptional changes as cells enter the exhaustion state, and so is potentially mediated in some fashion by similar factors such as TOX, BATF, or BLIMP-1.

7. "The authors should state the statistical analysis used in each legend."

We have corrected this oversight by providing additional details on the statistical analysis used, and thank the reviewer for their constructive attention to detail. .

Reviewer #2:

"The manuscript entitled "Multiomic Profiling of Chronically Activated CD4+ T Cells Reveals Novel Drivers of T-Cell Exhaustion Phenotype and Metabolic Reprogramming" explores a significant gap in our knowledge of exhaustion. CD4 T cells orchestrate the response of multiple cell types, including dendritic cells, and have a significant role in the overall effectiveness of the immune response. Understanding how CD4 T cell exhaustion influences the immune response against chronic infections and cancer is of vital importance for increasing our ability to intervene and improve patient outcomes. The manuscript provides significant advances in our understanding but could benefit from the following suggestions."

We thank the reviewer for their positive, astute, and constructive feedback.

Major critiques:

1. *The authors base their results on an in vitro model of exhaustion. However, while these T cells might express the hallmarks of exhaustion (such as co-inhibitory marker expression), this method cannot recapitulate all the signals present in vivo. This paper would benefit from in vivo validation.*

This is a valid point. To address the need for independent validation, we have performed and include extensive additional *in vivo* verification analyses, as summarized below, including flow cytometric analysis of HIV+ patient T cells that verify the elevated surface abundance of novel receptors identified in our study (CD276 and FLT1 now shown in Figure 4 & Supplemental Figure 7), confirmed increased expression of T cell exhaustion markers in both CD8+ and CD4+ T cells in breast cancer tumors based on single-cell sequencing (Figure 7, shown below), and proteomic analysis of pooled HIV+ donor T cells that primarily supports observed metabolic shifts (Table 1 and Table 2, shown below).

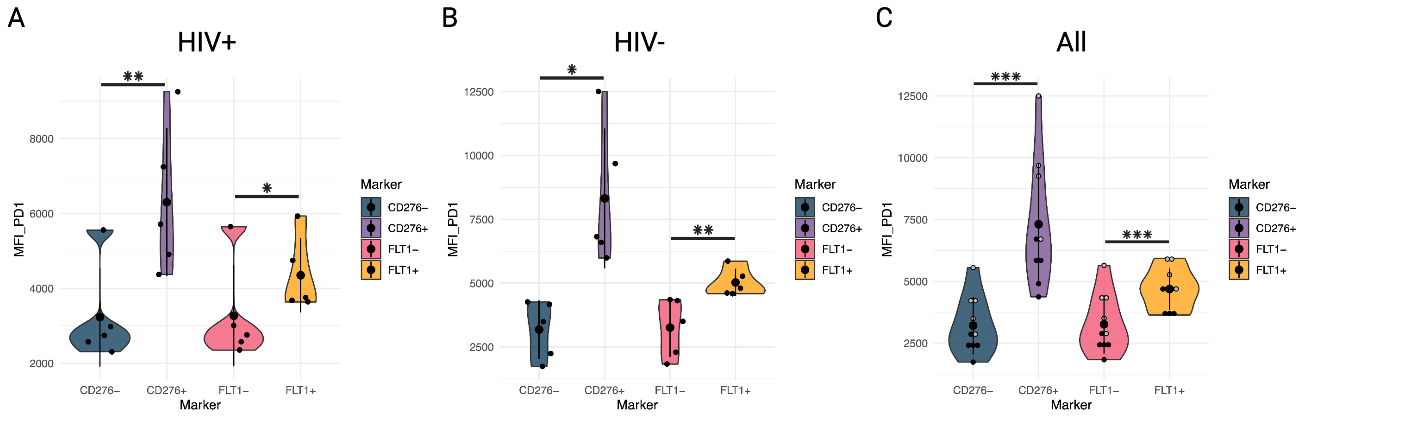

Figure 4. **Flow cytometric analysis of HIV+ and HIV- primary T cells reveals preferential high co-expression of PD-1 on CD276+ and FLT-1+ CD4+ T cells.** Plots shows mean fluorescence intensity (MFI) of PD-1 recorded for CD4+ T cells obtained from (A) HIV+, (B) HIV-, or (C) combined patients that are CD276+ or FLT-1+.

**Metabolic pathways enriched in IR+ vs IR- HIV+ donor T cells and day 6 vs resting *in vitro* exhaustion model**	**Metabolic pathways enriched in IR+ vs IR- HIV+ donor T cells and day 12 vs resting *in vitro* exhaustion model**
ADENINE AND ADENOSINE SALVAGE VI	*ADENINE AND ADENOSINE SALVAGE VI*
GDP-MANNOSE BIOSYNTHESIS	*GDP-MANNOSE BIOSYNTHESIS*
GALACTOSE DEGRADATION I	*GALACTOSE DEGRADATION I*
GLYCOGEN DEGRADATION II	*GLYCOGEN DEGRADATION II*
HYPUSINE BIOSYNTHESIS	*HYPUSINE BIOSYNTHESIS*
ADENINE AND ADENOSINE SALVAGE I	*ADENINE AND ADENOSINE SALVAGE I*
RETINOATE BIOSYNTHESIS I	*RETINOATE BIOSYNTHESIS I*
ARGININE DEGRADATION I	*ARGININE DEGRADATION I*
CITRULLINE DEGRADATION	
<IMYO</I-INOSITOL BIOSYNTHESIS	

Table 1. MOMENTA predicts metabolic pathways enriched in HIV+ donor IR+ T cells that reflects many seen in in vitro exhausted cells
MOMENTA was used to perform metabolic pathway enrichment of HIV+ donor inhibitory receptor (IR) positive cells against IR negative cells. Shown in the table are significant metabolic pathways from this analysis that are also found in day 6 vs resting comparison of our dataset (left column) and day 12 vs resting of our dataset (right column).

**Pathway**	**p value**	**Odds Ratio**	**Database**
Prostaglandin biosynthesis and regulation	0.00001669	30.67	BioPlanet2019
Prostaglandin Synthesis And Regulation WP98	0.00007489	20.18	WikiPathway 2023
Hypoxia	0.003324	5.32	MSigDB Hallmark 2020
p53 Pathway	0.01807	4.19	MSigDB Hallmark 2020
Fatty Acid Metabolism	0.04489	3.94	MSigDB Hallmark 2020

Table 2. Key pathways highlighted in in vitro exhaustion model show enrichment in HIV+ donor IR+ T cells
Selected pathways from Enrichr of the top 100 genes upregulated in the comparison of IR+ versus IR- HIV+ donor T cells are shown here as confirmation that these pathways are seen both in our in vitro model as well as in vivo

2. *The authors examined most of their data in relation to other data sets available for CD8 T cell exhaustion. While this provides very useful to determine which genes are important in both CD4 and CD8 T cell exhaustion, this analysis does not allow for the exploration of genes that are only relevant for CD4 T cell exhaustion. For example, while CD40L is an extremely important way CD4 T cells are capable of communicating with other cells in the system, CD40L was never examined in the paper. This paper would benefit from some unbiased analyses of just the CD4 signatures, without comparing the data to CD8 data.*

This is a great point. We now expand on CD4+ specific proteins, including CD40L, in the updated Figure 2 and the revised Discussion.

3. *The authors have provided a great first pass at uncovering CD4 T cell exhaustion and the authors do an outstanding job presenting the data in the context of known (CD8) literature. However, the data presented in most of the paper are mostly descriptive in nature. For example, the authors see an increase in ATM and the implication is that inhibition of ATM has been shown restore CD8 T cell function. While this is true for CD8 T cells, this may not be a relevant pathway for CD4 T cells. This paper would benefit from functional validation for what these targets mean in the context of CD4 T cell exhaustion (similar to what was done in figure 4*[now figure 5]*).*

We appreciate the recognition of our paper and its presentation but acknowledge limitations. To address these concerns, as noted above, we performed a flow cytometric analysis of HIV+ patient T cells, which independently verified that elevated surface levels of two new markers we identified occurs on a subpopulation of CD4+ T cells in vivo that also have significantly increased PD-1 (now provided as Figure 4). Moreover, as shown above (and provided in Figure 7), we also now demonstrate that tumor resident CD4+ T cells express many of the other markers *in vivo* that we found in our study (including factors implicated in CD8+ dysfunction).

4. *PBMCs were expanded prior to use for mass spectrometry-based assays. As the stimulation methods are very similar to those used to generate and exhausted phenotype (CD3/CD28 stim) and the length of culture was similar (10 days), how does this influence the phenotypes observed in the manuscript?*

We apologize for any confusion and have now clarified the protocol. The key difference between exhausting versus expanding the cells is the extent of antigen (CD3/CD28 Dynabeads) presence during the time-course. For expansion (without exhaustion), T cells were stimulated for just 2-3 days, at which point the beads were removed; then the cells were allowed to proliferate normally. This is a well-established method for expanding T cells and is suggested by the bead manufacturer.

5. *The data presented in figure 4D and E* [now Fig 5D & E] *would be more interpretable if the individual data points were plotted. Additionally, as error bars are depicted, the authors should clarify whether these data points represent technical or biological replicates. If these data points are technical replicates, the statistical analysis is not valid, as differences in technical replicates are more likely to represent variability in technique rather than biological spread.*

We appreciate the constructive suggestions. The replicates were same donor but were treated individually in different cell culture wells and so were treated as independent biological experiments. The data formats shown in Fig 5 D and E are consistent with the literature with how MFI and differences in flow marker population are commonly shown, but we now include the actual flow cytometric files as supplemental material so that interested readers can see individual sample values.

<u>*Minor critiques:*</u>

1. *The authors started with CD45RO+ CD4 T cells. It is unclear why the authors investigated an exhausted phenotype in the memory fraction of CD4 T cells compared to the acquisition of this phenotype by naïve T cells.*

We opted to analyze memory CD4+ T cells to reduce heterogeneity of the starting cell population to a practical degree, while still maintaining enough cells to perform in depth multi-omic profiling. We also reasoned that memory subtypes are more frequent progenitor population of cells found in tumors, and have now expanded upon our selection criteria in the updated manuscript.

2. *As CD4 T cells have an important role as "helper T cells", please provide data of CD40L expression over time to validate your model (fig 1).*

We appreciate the reviewer noting this important point, and now highlight CD40L plus other CD4+ related proteins in updated Figure 2.

3. *The data presenting average cell divisions (1D) and coinhibitory marker expression (1E) appear to represent a single data point. Please provide additional data to ensure reproducibility.*

While a single data point is shown in Figure 1, we now include additional supporting data in Supplemental Figure 1.

4. *As increased apoptosis is a feature of the progression of CD8 T cell exhaustion, including T cell viability and apoptosis data would be beneficial.*

To demonstrate cell death present in these cultures, Zombie NIR Live/Dead staining in triplicate from donor #2 is shown on the next page and included as an additional supplemental figure (Supplemental Figure 2). This live/dead percent is based on gating of the entire collected sample without any previous gating. With the exception of early activation conditions (day 2 to day 6), cell cultures mostly stayed around ~85% viability.

Live/Dead Staining (Zombie NIR)

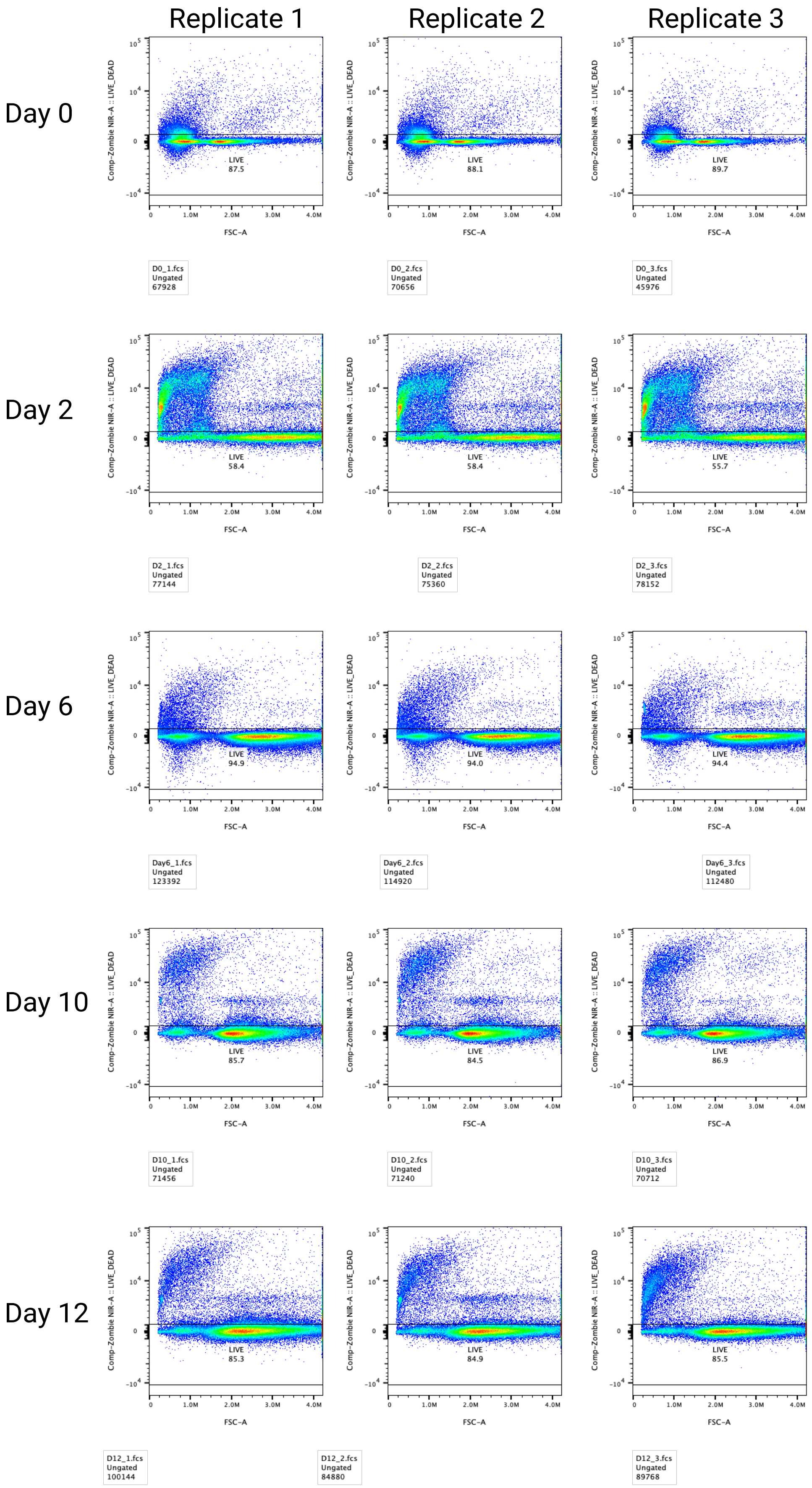

5. *Fig 3: the light green and yellow are hard to distinguish.*

   The color/saturation has been adjusted for better contrast.

6. *Fig 4D* [now Fig 5D]*: The authors mention in the text that there are more cells with lower expression of inhibitory markers. However, this data was not was presented. Please include the appropriate graphs (frequency and absolute numbers) to support this statement.*

   We would like to clarify our data representation. The y-axis on figure 5D shows cell number (normalized to mode to account for slight differences in the number of cells collected/analyzed per sample, but still allows for population comparison). We show that more cells (by % in population) have lower expression of PD-1 and TIM-3 when treated with p300 inhibitor. However, we now include the requested stats (frequency and absolute numbers) in the updated figure legend.

7. *Fig 4E* [now Fig 5E]*: The authors write in the text that PD1/TIM3 expression was not equivalent to resting or Dasatinib treated samples. However, there are no statistical significant changes depicted in the graphs so this statement is not representative of the data depicted.*

   We have added the requested statistical significance to Figure 5E to properly back up this claim.

8. *Fig 4E* [now Fig 5E]*: If there is a statistical difference between DMSO and p300 inhibition, there is likely also a difference between DMSO and tested and Dasatinib treatment. It is unclear why the authors only showed the significant difference between DMSO and p300 inhibition. It is also unclear what statistical method the authors used, as no statistical analysis information was provided in the method section of the paper.*

   As noted above, we have updated the figure (plus legend) to establish statistical. Significance in Fig. 5E was obtained by via T-test (performed using R).

9. *Fig 4F* [now Fig 5F]*:: CASCADE analysis: the entire paper compares rested or day 6 stimulated samples to late time points (d10 or d12). It is unclear why the authors chose to compare day 2 samples to day 12 samples for this analysis.*

   We were only able to analyze day 2 vs 12 by CASCADE due to assay throughput limitations. We believe this is a critical comparison as it directly compares the response to antigen challenge of healthy responsive T cells to those in a chronically stimulated, exhausted state.

Reviewer #3:

"The authors utilize quantitative proteomic, phosphoproteomic, and metabolomic analyses to characterize the molecular basis of the dysfunctional cell state induced by chronic stimulation of CD4+ memory T cells. They focus on CD4+ T cells to avoid confounding influence of T cell heterogeneity by CD8, DN. While multiple and granular studies, albeit not proteomic, have highlighted and detailed exhaustion in CD8 T cells, this is the first proteomic study in exhausted CD4+ T cells. Therefore, I find this investigation timely and necessary for few reasons: i) To my knowledge this is the first quantitative phospho/proteomic and metabolomic resource of exhausted human CD4+ T cells; ii) Integrative analysis validates and highlights targetable kinases in CD4 Tc exhaustion; iii) Integrative analysis identifies p300 as mediator of CD4 exhaustion and validates that p300 inhibition decreases the expression of PD1 and TIM3 inhibitory receptors; iv) the findings of this investigation have potential to inform additional therapeutic approaches for re-invigoration/targeting of exhausted CD4+ T cells in chronic inflammation. My comments below are, in my opinion, addressable by the authors."

We thank the reviewer for their positive, constructive feedback.

Major concerns:

1. *My major concern about the paper narrative is that clarifications are required in the main text in few occasion to guide the reader through the rigor of the QC process related to the protocol of exhaustion, number of donor samples and global proteomic reproducibility of the second donor sample. With regard to figures, while computational analysis is well presented, some annotations are lacking and I have described them below. Additionally, n=1 is presented for majority of QC studies related to exhaustion protocol, and all donors used in the QC process and during development of the assay should be presented the demonstrate reproducibility and support claims.*

We appreciate the critique, and recognizing that our sample size is limited, that the data from donor 2 was not well displayed. To better show the consistency between the two independent experiments, we now highlight the results obtained for donor #2, both below and in Supplemental Figure 1, which includes both protein expression detected via quantitative mass spectrometry as well flow cytometry data for surface markers. We have also updated the main text to better clarify the large overall consistency seen between our two separate analyses.

Total Protein Identification Overlap

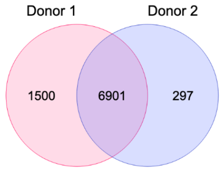

Please note time points are not identical in each donor, but do mostly follow the same pattern based on matching time points.

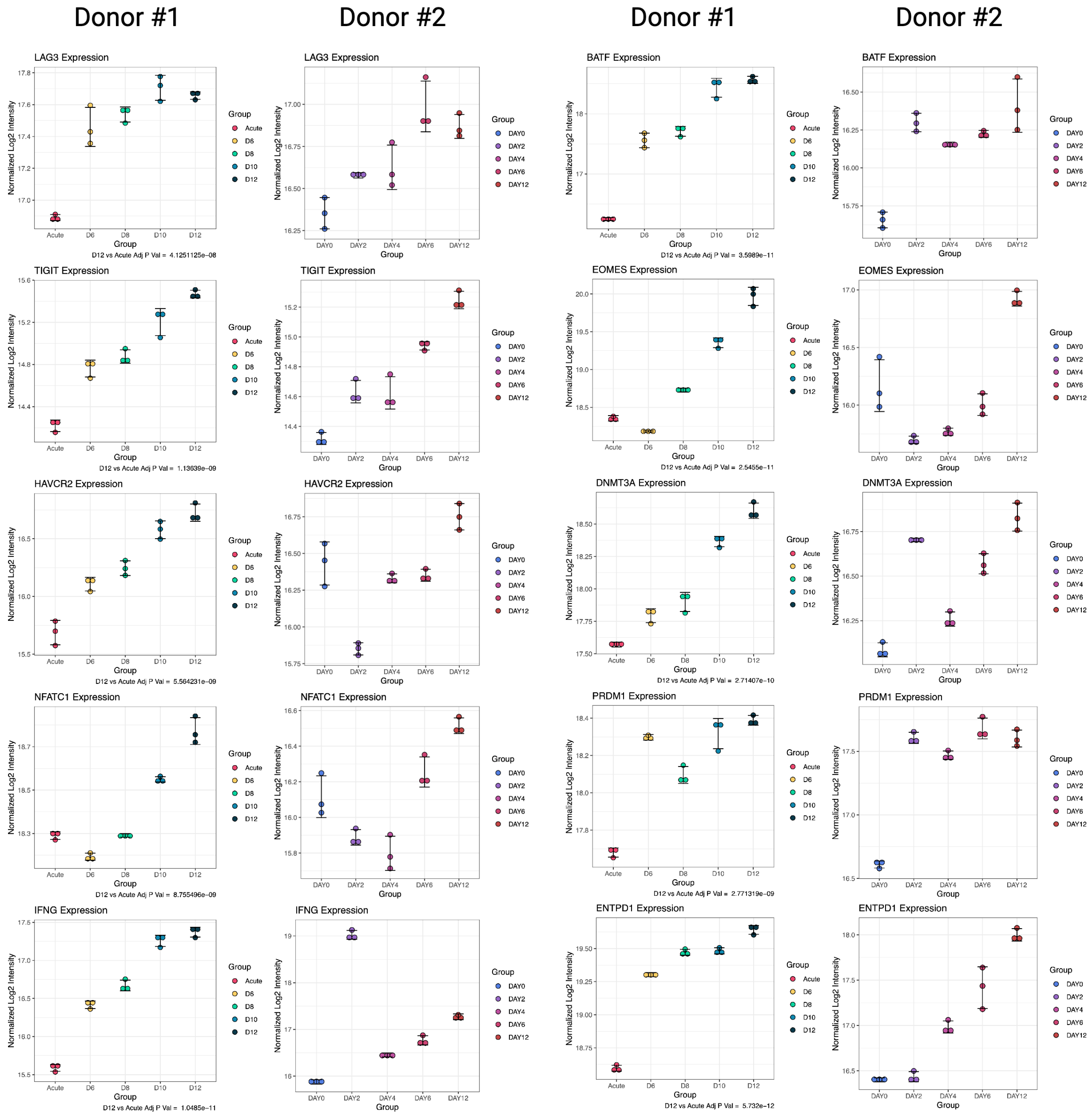

2. *Also, please cite necessary references relevant to the exhaustion protocol.*

Thank you for noting this oversight. Updated references have now been inserted at the correct lines (references 50-54 in revised manuscript numbering). References included are below (1-5).

1. Dunsford, L. S., Thoirs, R. H., Rathbone, E., & Patakas, A. (2020). A Human In Vitro T Cell Exhaustion Model for Assessing Immuno-Oncology Therapies. In S.-L. Tan (Ed.), *Immuno-Oncology: Cellular and Translational Approaches* (pp. 89–101). Springer US. https://doi.org/10.1007/978-1-0716-0171-6_6
2. Balkhi MY, Wittmann G, Xiong F, Junghans RP. YY1 Upregulates Checkpoint Receptors and Downregulates Type I Cytokines in Exhausted, Chronically Stimulated Human T Cells. iScience. 2018 Apr 27;2:105–22.
3. Simula L, Antonucci Y, Scarpelli G, Cancila V, Colamatteo A, Manni S, et al. PD-1-induced T cell exhaustion is controlled by a Drp1-dependent mechanism. Molecular Oncology. 2022;16(1):188–205.
4. Zhao M, Kiernan CH, Stairiker CJ, Hope JL, Leon LG, Meurs M van, et al. Rapid in vitro generation of bona fide exhausted CD8+ T cells is accompanied by Tcf7 promotor methylation. PLOS Pathogens. 2020 Jun 24;16(6):e1008555.
5. Zhu L, Zhou X, Gu M, Kim J, Li Y, Ko CJ, et al. Dapl1 controls NFATc2 activation to regulate CD8+ T cell exhaustion and responses in chronic infection and cancer. Nat Cell Biol. 2022 Jul;24(7):1165–76.

3. *With regard to used references: in multiple occasion, the authors cite review papers rather than original research. An examples is line 99 (ref 21)-the authors can cite original scRNAseq of CD4+ T cell exhaustion by Camille-Charlotte Balança et al (JCI, 2020). Additionally, they should include an important references describing CD4+ T cell exhaustion in tumor infiltrating T cells by scRNAseq. Not all references follow the same format.*

We thank you the reviewer for pointing this out. We have updated the reference, citing specific primary research in multiples places, and have eliminated discrepancies in our citation formats.

4. *Authors used two independent donors and carried the analysis in triplicate. It is unclear if flow cytometry analysis, ELISA and cell viability were paired with the phospho/proteomic experiments.*

We apologize for the confusion. All experiments were paired to donor #1 unless specified, e.g. the newly expanded cytokine panel shown in the updated supplemental material is paired with donor #2.

5. *Unclear how many donors were used to QC the exhaustion protocol and exhaustion marker expression. Only one donor shown in sup figure 1 and second in sup figure 3, but necessary to be confirmed across multiple donors (3-4 donors) for protocol reproducibility.*

We provide 2 independent donor replicates in the paper, but note that during testing, several other donor cells responded similarly to chronic stimulation, as noted before by Dunsford et al, Balkhi et al, and Simula et al, among others (1-5 below):

1. Dunsford, L. S., Thoirs, R. H., Rathbone, E., & Patakas, A. (2020). A Human In Vitro T Cell Exhaustion Model for Assessing Immuno-Oncology Therapies. In S.-L. Tan (Ed.), *Immuno-Oncology: Cellular and Translational Approaches* (pp. 89–101). Springer US. https://doi.org/10.1007/978-1-0716-0171-6_6
2. Balkhi MY, Wittmann G, Xiong F, Junghans RP. YY1 Upregulates Checkpoint Receptors and Downregulates Type I Cytokines in Exhausted, Chronically Stimulated Human T Cells. iScience. 2018 Apr 27;2:105–22.
3. Simula L, Antonucci Y, Scarpelli G, Cancila V, Colamatteo A, Manni S, et al. PD-1-induced T cell exhaustion is controlled by a Drp1-dependent mechanism. Molecular Oncology. 2022;16(1):188–205.

4. Zhao M, Kiernan CH, Stairiker CJ, Hope JL, Leon LG, Meurs M van, et al. Rapid in vitro generation of bona fide exhausted CD8+ T cells is accompanied by Tcf7 promotor methylation. PLOS Pathogens. 2020 Jun 24;16(6):e1008555.
5. Zhu L, Zhou X, Gu M, Kim J, Li Y, Ko CJ, et al. Dapl1 controls NFATc2 activation to regulate CD8+ T cell exhaustion and responses in chronic infection and cancer. Nat Cell Biol. 2022 Jul;24(7):1165–76.

6. *With regard to p300 validation, it is clear that inhibition reduces the expression of PD1 and TIM3, however in supplement the authors show that it also increases LAG3 and TIGIT expression, which will have consequences in chronic inflammation. I would encourage the authors to elaborate on this in the discussion and its potential implications.*

We appreciate this valid point, and now elaborate on the potential consequences of up (and down) regulation of different inhibitory factors into our updated Discussion.

<u>Line specific critique responses:</u>

Line 116-no reference to established culture model

We thank the reviewer for spotting this oversight; culture model references have been added (see response #2 above for specific references).

Line 145: Please clarify in main text that prior to carrying out chronic exhaustion, CD4 T cells were polyclonally expanded with anti-CD3/CD28.

The cells were expanded before use, and the text has been updated to clarify this for readers.

Figure 1C-please clarify number of samples and what is represented in error bars.
Are all of the data paired-i.e. inhibitory receptor expression, cell division and cell death were confirmed in the same sample that was subjected to proteomics?

Three replicates were used, and figure legend was updated to reflect that. Samples were paired.

Figure 1D-include all donors in the figure to demonstrate reproducibility. Additionally, line 163 states minor cell death, but sup figure 1 SSC-A vs. FSC-A suggests ~50% cell death. Was that replicated across donors and time points?

We thank the reviewer for noting this, and we have suitably updated the text. The cells do show some cell death during the early stimulation and expansion, which is commonly seen when expanding T or stimulating cells with Dynabeads. The cell death stabilizes, before rising slightly again as cells enter later stages of chronic activation. The text has been updated to reflect this, as has the supplemental material to show conditions with less cell death than reported before for day 6.

Figure 1E-show second donor for reproducibility (also include in sup figure 1). Or include all donors used for QC in bargraphs

The second donor flow cytometry is now included with the updated manuscript (Supp. Figure 1).

Supp figure 1 [now Supp Fig 4]. *Were the observed changes in CD4 inhibitory receptor upregulation consistent and QC'ed across multiple donors undergoing this exhaustion protocol. Dot plot flow axis should be transformed to include all events.*

Upregulation of inhibitory receptors on CD4+ T cells was seen on <u>both</u> donors, as well as other test runs for separate experiments not reported in this paper. As stated previously, others have used this same protocol and observed similar outcomes (references listed in response #2).

Supp figure 3. It will be greatly beneficial to show reproducibility of the global protein expression by including how many total proteins were identified and shared between the two donors. Were exhaustion marker panels shown in figure 2 reproducible in second donor?

A Venn diagram is now added to Supplemental Figure 5 to show dataset protein overlap (7,000 total proteins crossmap between both datasets). Despite some modest variation, which is expected between different donors, overall the exhaustion markers shown in Figure 2 were largely in agreement. Expression profiles of markers are shown either in response #1 figure and/or as new Supplemental Figure 1, and all expression values are provided in Supplemental File 1. We note that some proteins were not detected in both datasets, for example PD-1 was not detected in donor #2. In proteomics, this may not reflect low levels. Rather, receptors and other membrane proteins tend to be more variable for detection due to challenges with solubility during sample preparation.

Line 827: Please clarify when were last Dynabeads added day 8 or 10?

Final beads were added on day 10 of 12-day protocol. Methods are now updated and clarified.

Line 144-146: It is unclear that these are pre-expanded T cells, which is needed to obtain sufficient numbers, and clarification is necessary as not to confuse the reader.

Thank you, this has been clarified in both the revised main text and updated methods section to make it clear cells were expanded first, then brought to resting before undergoing exhaustion protocol.

Figure 2: please indicate what is shown in heatmap (row z-score?).

Yes, heatmaps were row z-scored normalized; legend and figure now updated to reflect that.

Figure 2E-please include examples of specific proteins: i.e.-since cell cycle clusters overlap between activation/intermediate chronic and chronic activation/exhaustion, it is important to clarify which are the proteins in cell cycle that are different between the two groups. Same for calcineurin-regulated NFAT-dependent transcription shared between group 2 and 4. Also, line 264-authors refer to NFAT-related proteins, please indicate which ones. Same for line 275-please indicate key representative examples of proteins that are downregulated to negatively control activation.

These are excellent points, and appropriate changes have now been made (as noted here and further below). The cell cycle geneset is large, as are the number of protein hits from each cluster within this geneset. Instead of listing them in the manuscript itself, we have generated another supplemental file for reference to the genes from each.

Proteins from calcineurin-regulated NFAT-dependent transcription geneset from each cluster has been added to differentiate the two. As a note, it appears that most follow a pattern of being upregulated by day 12, but are split based on whether they are also high while resting or not, implying strongest signal comes from day 12 as a whole.

The NFAT proteins referred to are now listed in that line.

Examples representative of downregulated proteins that regulate T cell activation include IL2RB, CD3E/CD3G, LCK, CD4, and OXPHOS proteins such as those in the ATP synthase complex (eg ATP5B/E/I).

Line 280, include original reference.

Original reference has now been added.

Supp figure 4, lines 367-371-include examples of proteins with heavily phosphorylated phosphosites in resting cells.

We have added certain proteins for each pathway mentioned from gene sets showing increased phosphorylation in the resting condition.

Figure 2G: 1) while expected that majority of sites will be pS/pT, it will be beneficial to show what is the number of detected total pY/S/T sites; 2) how many are shared vs time point specific; 3) how does protein expression throughout exhaustion of respective kinases influence kinase-substrate relationships- i.e. does kinase down/up-regulation influence phosphorylation patterns.

Bar chart showing pS/Y/T site counts has now been added to the supplemental phospho figure (shown below). Quantitation was based on tandem mass tags (TMT), which exhibit dynamic range compression, but based on the number of adjusted p values < 0.05 for every comparison of each time point to each other (10 total possible comparisons), we see that 954 phosphosites have only 1 single comparison that is statistically significant (see table below), meaning 954 phosphosites are time point specific.

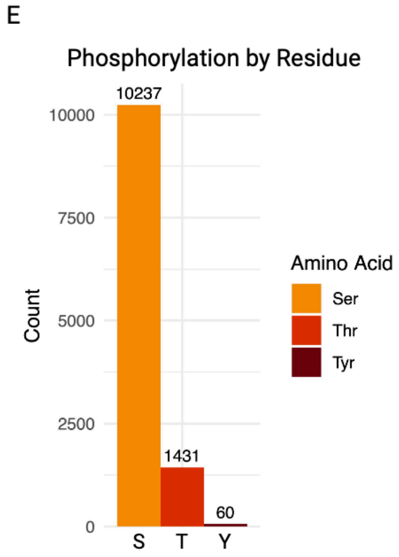

# of Adj. p vals < 0.05	# of phos sites
0	2527
1	945
2	950
3	965
4	3417
5	1066
6	967
7	506
8	2752
9	30
10	2

As the protein levels of kinases is not a reliable indicator of altered kinase function, we have not emphasized this data, rather we instead investigate kinase and substrate phosphorylation, which is deemed more reliable indicator of enzymatic function. Notably, a substantive subset of the kinases implicated in our KSEA analysis do not show a significant alteration in protein abundance.

Figure 3: 1) y axis numbers are missing. 2) Line 417-please elaborate which associations the authors are referring to. 3) Line 419-421-in that case will be beneficial to perform integrative analysis to identify whether these proteins are also substrates for the identified kinases, or from literature searches whether their expression is regulated by those kinases.4) Main text related to figure 3B is not clear as to which cluster the authors are referring to.

1. The Y-axis was intentionally left blank as the Y-axis is intensity values (as described in figure), which vary for each protein. The main purpose of displaying these proteins expression plots is not the exact intensity value, but rather the expression pattern over time.
2. Text added specifying which exhaustion proteins were found each cluster, as now displayed in the updated figure.

3. This is an excellent suggestion, but current mapping of kinase-substrate interactions is still limited. Nevertheless, we attempted an integrative analysis (to link transcription factors and cell surface proteins in the clusters displayed in figure 3) to kinases following similar patterns. We have included some of our findings , which include NFYB, a transcription factor in Fig. 3B linked to 11 of 21 of kinases in this cluster, and TCF3, connected to another 5. The analysis was performed by taking kinases in the cluster that is upregulated over time (cluster 3), and using them as input for X2K analysis (Expression2Kinases, Ma'ayan lab). This provides predicted upstream regulators of the kinases given, and we get 2 hits that are also found in the transcription factor upregulated clusters shown in figure 3B.
4. The intent of showing 2 clusters in Figure 3B reflects their profile similarity (upregulation in chronically stimulated cells), and the prevalence of known exhaustion-associated transcription factors (e.g. TOX, BLIMP-1, NAFTs, etc.) in both. There may be other transcription factors related to exhaustion of CD4+ T cells present, as noted for Fig. 3A.

Line 515-516: missing reference.

We now cite references linking non-canonical NFkB signaling to Blimp-1 expression via RelB.

Line 619: give examples of enzymes changes linked to metabolic pathways.

The main text was adjusted for clarity on this point.

Line 266-why is line in quotations and italicized-is this a citation? If so, please include reference.

We apologize for this inadvertent formatting error.
